# Medicaid Accountable Care Organization Implementation and Perinatal Claims Documentation of Social Risk Factors

**DOI:** 10.1001/jamanetworkopen.2025.5999

**Published:** 2025-04-21

**Authors:** Kevin H. Nguyen, Sarah H. Gordon, Kenneth Lim, Kathryn D. Thompson, Collette N. Ncube, Megan B. Cole

**Affiliations:** 1 Department of Health Law, Policy, and Management, Boston University School of Public Health, Boston, Massachusetts; 2 Department of Community Health Sciences, Boston University School of Public Health, Boston, Massachusetts; 3 Department of Epidemiology, Boston University School of Public Health, Boston, Massachusetts

## Abstract

**Importance:**

Addressing social risk factors (eg, food insecurity) during the perinatal period has the potential to improve pregnancy-related outcomes. While social risk factor diagnosis codes (ie, *International Statistical Classification of Diseases, Tenth Revision,* Z codes) were introduced in 2016, adoption in claims has been slow. In 2018, Massachusetts’ Medicaid program implemented an accountable care organization (ACO) model, including a requirement that all ACOs screen for social risks.

**Objective:**

To assess claims documentation of Z codes in the perinatal period for Medicaid enrollees and evaluate changes in documentation following implementation of Massachusetts’ Medicaid ACO program.

**Design, Setting, and Participants:**

This cross-sectional study used the Massachusetts All-Payer Claims Database to identify all Medicaid-enrolled live deliveries between January 31, 2016, and December 31, 2020, among people 18 years or older. A difference-in-differences (DiD) approach was used to compare Z code documentation before (2016-2017) vs after (2018-2020) ACO implementation for Medicaid ACO vs non-ACO deliveries. Data were analyzed between August 23, 2024, and January 27, 2025.

**Exposures:**

Attribution to a Medicaid ACO (vs non-ACO), determined based on whether an enrollee’s primary care physician participated in an ACO.

**Main Outcomes and Measures:**

The primary outcome was claims documentation of any Z code, measured separately for the prenatal period, 60 days post partum, 12 months post partum, and the full perinatal period. Secondary outcomes included documentation of specific Z codes (eg, housing, food insecurity).

**Results:**

The study sample included 79 293 deliveries (mean [SD] age of Medicaid-enrolled pregnant people, 28.2 [5.7] years), of which 69 535 (87.7%) were in a Medicaid ACO. Among all Medicaid deliveries from 2016 to 2020, 4.45% had claims documentation of a Z code in the prenatal period; 1.14%, within 60 days post partum; and 3.31%, within 12 months post partum. Within the prenatal period, Medicaid ACO implementation was associated with statistically significant increases in documentation of any Z code (DiD, 1.09 [95% CI, 0.38-1.80] percentage points [PP]), housing or economic circumstances codes (DiD, 1.52 [95% CI, 1.07-1.97] PP), and food insecurity codes (DiD, 0.58 [95% CI, 0.42-0.73] PP). The Medicaid ACO was associated with few changes in Z code documentation in the postpartum period.

**Conclusions and Relevance:**

In this cross-sectional study of Medicaid-enrolled live births in Massachusetts, ACO implementation was associated with increased claims documentation of Z codes during the perinatal period, driven by increases in the prenatal period. While Z code documentation remains low, implementation of care delivery models that incentivize screening and documentation of social risk factors among Medicaid enrollees may help to identify the care needs of pregnant and postpartum people.

## Key Points

**Question:**

How were social risk factor–related diagnosis codes (ie, Z codes) documented among pregnant Medicaid enrollees following Medicaid accountable care organization (ACO) implementation in Massachusetts?

**Findings:**

In this cross-sectional study of 79 293 deliveries, Medicaid ACO implementation was associated with modest, statistically significant increases in Z code documentation, particularly in the prenatal period. Increases were largest for documentation of codes related to housing or economic circumstances.

**Meaning:**

These findings suggest that, although documentation of social risk factors remained relatively low, requiring social risk factor screening as part of an ACO contract may increase use of Z codes among perinatal populations.

## Teaser

This cross-sectional study evaluates documentation of social risk factor–related Z codes in the perinatal period for Medicaid enrollees and changes in documentation rates following the implementation of Massachusetts’ Medicaid accountable care organization (ACO) program.

## Introduction

Medicaid finances more than 40% of births in the US and plays a crucial role in the health and well-being of people during pregnancy and post partum and of infants.^1^ Evidence suggests that pregnant and postpartum Medicaid enrollees often experience poor health outcomes across the perinatal period.^1,2^ Some states have implemented large-scale care delivery reforms to improve access, quality, value, and equity of care for Medicaid enrollees, some of which could potentially benefit pregnant and postpartum people. For example, as of 2024, 12 states implemented Medicaid accountable care organizations (ACOs), a form of value-based care wherein practices, hospitals, and clinicians agree to take responsibility for the cost and quality of care delivered to an attributed patient population.^2,3^ Given the growing recognition of the role of an individual’s social circumstances in health and well-being, some state Medicaid ACOs encourage or require interventions related to social risk factors (eg, food insecurity, housing instability), which are individual-level adverse conditions experienced by a person that can negatively impact their health and well-being.^4,5^

Among pregnant people specifically, screening for social risk factors and addressing unmet health-related social needs (ie, a social risk factor for which a patient would like assistance) during the perinatal period has the potential to improve pregnancy-related outcomes.^6,7^ The prenatal period, a high-contact period with health care professionals, may provide several opportunities to screen for and address unmet health-related social needs.^7^ Evidence has linked various social risk factors with adverse perinatal outcomes among the pregnant and postpartum population.^8^ For example, experiencing housing instability and homelessness during pregnancy was associated with preterm birth, delivery complications, and increased complications,^9,10^ while experiencing food insecurity was associated with stress, anxiety, and depression during pregnancy.^11^ Alternatively, screening for and intervening in unmet health-related social needs (eg, through referral to social services) has been associated with reduction in preterm birth.^12^ Though there is substantial evidence about increases in screening and documentation of social risk factors across clinical settings over the last decade, little is known about documentation of social risk factors throughout the perinatal period, particularly among pregnant people enrolled in Medicaid.^13,14,15,16,17^

In 2018, through a Section 1115 demonstration waiver, Massachusetts’ Medicaid program implemented an ACO model to better coordinate and integrate physical, behavioral, and social needs of enrollees, including a requirement that all ACOs screen for social risk factors and the use of risk-adjusted ACO payments that account for measures of unstable housing.^2,18,19,20,21,22^ Among health care delivery sites, one approach to documenting social risk factors is through diagnosis codes (ie, *International Statistical Classification of Diseases, Tenth Revision,* Z codes), which were introduced in 2016. Although evidence suggests that their adoption in administrative claims has been slow,^23,24^ the social risk screening requirements and the opportunity to apply Z codes toward risk adjustment and payment purposes through Medicaid ACOs may increase their adoption. To address this gap, our study evaluated documentation of social risk factor–related Z codes in the perinatal period for Medicaid enrollees and evaluated changes in documentation rates following the implementation of Massachusetts’ Medicaid ACO program.

## Methods

### Study Setting

Through a Section 1115 demonstration waiver in Massachusetts, 17 ACOs were formed under a statewide Medicaid ACO program beginning in March 2018. For all attributed Medicaid enrollees, each ACO was responsible for the cost, quality, and member experience. One of the 20 contractual ACO pay-for-performance metrics included the percentage of ACO members who were screened for health-related social needs within the measurement year, as measured through both electronic health record and claims data. To support this measure, ACOs were required to implement a social risk factor screening tool and, when appropriate, link members to social service organizations. Additionally, to calculate financial targets for ACOs, a risk-adjustment model was developed to account for both traditional medical factors (eg, diagnoses) and social factors (eg, unstable housing and neighborhood-level variables).^20,21,25^ Accounting for unstable housing in ACO payments aimed to minimize incentives to limit care or avoid people with greater care needs.^19^ The institutional review board at Boston University, Boston, Massachusetts, deemed the study exempt and waived informed consent because only deidentified data were used. We followed the Strengthening the Reporting of Observational Studies in Epidemiology (STROBE) reporting guidelines for cross-sectional studies.

### Data Sources and Study Sample

We used 2014-2020 data from the Massachusetts All-Payer Claims Database (APCD) to identify all Medicaid-enrolled live deliveries occurring between January 31, 2016, and December 31, 2020, among members 18 years and older with at least 60 days of prenatal and postpartum Medicaid enrollment. The Massachusetts APCD includes member enrollment files, medical claims, and pharmacy claims for all Medicaid-enrolled people and most privately insured people in Massachusetts. Secondary data sources included the 2018-2020 Massachusetts Registration of Provider Organization files, which were used to link APCD National Provider Identifiers to ACOs; the Massachusetts Medicaid directories, which were used to attribute clinicians and practices to ACOs; and the 2018 American Community Survey data, which were used for zip code–level demographic characteristics. These included racial or ethnic composition and median household income; racial and ethnic composition were added because of evidence they are associated with birth outcomes.

### Assignment to ACO vs Non-ACO Groups

Deliveries were attributed to a Medicaid ACO or non-ACO using an algorithm described in previous work.^2^ Specifically, each Medicaid-enrolled live delivery was attributed to a primary care clinician with whom the member had the most recent primary care visit. The delivery was assigned to the Medicaid ACO intervention group if the attributed primary care physician was part of the Medicaid ACO as of 2018. The delivery was assigned to the non-ACO comparison group if attributed to a primary care physician who did not participate in the Medicaid ACO as of 2018.

### Main Measure

Our primary outcome consisted of claims documentation of any social risk factor–related Z code (eTable 1 in Supplement 1), measured separately for the prenatal period, 60 days post partum, 12 months post partum, and the full perinatal period. Secondary outcomes included documentation of specific social risk Z codes (eg, housing or economic circumstances, psychosocial circumstances, food insecurity, other) (eTable 1 in Supplement 1).

### Statistical Analysis

The unit of analysis was the delivery quarter. We first compared characteristics of ACO and non-ACO Medicaid-enrolled deliveries using Pearson χ^2^ tests for categorical variables (eg, delivery type, multiple gestation, documentation of ≥3 comorbid conditions, residence in a rural zip code, and previous diagnosis of select clinical conditions) and unpaired *t* tests for age at delivery (in years) and zip code–level continuous variables (eg, racial or ethnic composition, median household income).

We described overall social risk–related Z code documentation rates in the prenatal period, 60 days post partum, 12 months post partum, and the full perinatal period. We then used a difference-in-differences (DiD) design with linear probability models to compare Z code documentation before (2016-2017) vs after (from the third quarter of 2018 to 2020) ACO implementation for Medicaid ACO vs Medicaid non-ACO deliveries. We excluded the first 2 quarters of 2018 to account for the transitional Medicaid ACO implementation period.^2^ Models included an indicator for being in the ACO intervention vs non-ACO comparison groups, time (preintervention vs postintervention), and their interaction (ACO × postintervention). Models were adjusted for age, delivery type, multiple gestation, documentation of 3 or more clinical comorbidities, and zip code–level rurality and sociodemographic characteristics. Models included delivery hospital–level fixed effects, with SEs clustered at the individual level to account for repeated deliveries. Statistical significance was based on 2-sided *P* < .05.

We conducted multiple sensitivity analyses to assess the robustness of our findings. First, the DiD study design assumes that, in the absence of ACO implementation, outcomes would have trended similarly between intervention (ACO) and comparison (non-ACO) groups, often referred to as the parallel trends assumption. We both visually and statistically evaluated preintervention parallel trends, as described in the eMethods in Supplement 1. Second, to account for potential changes in utilization and outcomes during the COVID-19 pandemic, we excluded data from 2020 in a sensitivity analysis. All analyses were conducted in Stata, version 18.0 (StataCorp LLC), and data analysis was conducted between August 23, 2024, and January 27, 2025.

## Results

Our study sample included 79 293 deliveries (mean [SD] age of Medicaid-enrolled pregnant people, 28.2 [5.7] years), of which 69 535 (87.7%) were in a Medicaid ACO and 9758 (12.3%) were in a non-ACO. Compared with deliveries in non-ACOs, individuals with deliveries in Medicaid ACOs were less likely to have 3 or more comorbid conditions (1632 [16.7%] vs 10 161 [14.6%]; *P* < .001) and less likely to live in rural zip codes (1113 [11.4%] vs 3146 [4.5%]; *P* < .001) (Table 1). Prevalences of most clinical diagnoses and rates of cesarean section deliveries were similar between the 2 groups.

**Table 1.** Characteristics of Medicaid-Insured Deliveries by Enrollment in a Massachusetts Medicaid ACO, 2016 to 2020

Characteristic	Medicaid group^a^		All (N = 79 293)
	Non-ACO (n = 9758)	ACO (n = 69 535)	
Age of Medicaid-enrolled pregnant people, mean (SD), y	27.8 (5.7)	28.2 (5.7)	28.2 (5.7)
Delivery type, No. (%)^b^			
Vaginal	6152 (66.6)	42 807 (65.6)	48 959 (65.7)
Cesarean section	3091 (33.4)	22 482 (34.4)	25 573 (34.3)
Multiple gestation, No. (%)	180 (1.8)	1193 (1.7)	1373 (1.7)
≥3 Comorbid conditions, No. (%)	1632 (16.7)	10 161 (14.6)	11 793 (14.9)
Rural zip code, No. (%)	1113 (11.4)	3146 (4.5)	4259 (5.4)
Mental health diagnosis (ever), No. (%)	4328 (44.4)	27 756 (39.9)	32 084 (40.5)
Diabetes (ever), No. (%)	345 (3.5)	2450 (3.5)	2795 (3.5)
Asthma (ever), No. (%)	1726 (17.7)	10 678 (15.4)	12 404 (15.6)
Hypertension (ever), No. (%)	794 (8.1)	5680 (8.2)	6474 (8.2)
Overweight or obesity (ever), No. (%)^c^	2308 (23.7)	19 270 (27.7)	21 578 (27.2)
Zip code–level characteristics, mean (SD)			
Hispanic, %	16.3 (15.7)	22.1 (21.7)	21.3 (21.2)
Non-Hispanic Black, %	8.6 (11.8)	13.2 (16.8)	12.6 (16.4)
Non-Hispanic White, %	68.1 (23.2)	56.4 (26.9)	57.9 (26.7)
Median household income, US $	81 784.92 (30 872.30)	77 159.32 (30 772.41)	77 728.20 (30 821.98)

Abbreviation: ACO, accountable care organization.

^a^ Unit of analysis was delivery. Differences by enrollment in Massachusetts Medicaid ACOs were measured using Pearson χ^2^ tests for categorical variables and unpaired *t* tests for zip code–level continuous variables. *P* < .001 for all comparisons except delivery type (*P* = .06), multiple gestation (*P* = .36), diabetes diagnosis (*P* = .95), and hypertension diagnosis (*P* = .92).

^b^ Owing to missing data, denominators are less than totals in column headings.

^c^ Overweight is defined as a body mass index of 25 to 40; obesity is a body mass index greater than 40.

Among all Medicaid deliveries occurring in 2016 to 2020, 4.45% had any claims documentation of a social risk–related Z code in the prenatal period, 1.14% in the 60-day postpartum period, 3.31% in the 12-month postpartum period, and 6.84% in the perinatal period overall (eTable 2 in Supplement 1). In adjusted models, the Medicaid ACO in Massachusetts was associated with significant increases in documentation of any social risk–related Z code (DiD, 1.09 [95% CI, 0.38-1.80] percentage points [PP]; *P* = .003), housing or economic circumstances codes (DiD, 1.52 [95% CI, 1.07-1.97] PP; *P* < .001), and food insecurity codes (DiD, 0.58 [95% CI, 0.42-0.73] PP; *P* < .001) in the prenatal period (Figure, Table 2, and eFigures 1-3 in Supplement 1). Similar but larger associations were observed when assessing the entire prenatal and postpartum period: there were significant increases in documentation of any Z code (DiD, 1.49 [95% CI, 0.62-2.35] PP; *P* = .001), housing or economic circumstances codes (DiD, 1.88 [95% CI, 1.31-2.44] PP; *P* < .001), and food insecurity codes (DiD, 0.70 [95% CI, 0.48-0.93] PP; *P* < .001). The Medicaid ACO was associated with few statistically significant changes in Z code documentation during the 60-day and 12-month postpartum periods: the Medicaid ACO was associated with a relative increase in the probability of having a Z code for housing or economics circumstances in the 60-day postpartum period of 0.26 PP (95% CI, 0.002-0.51 PP; *P* = .048) and in the 12-month postpartum period of 0.50 PP (95% CI, 0.10-0.91 PP; *P* = .02). However, in both the 60-day and 12-month postpartum periods, there was no association between the Medicaid ACO and having documentation of any social risk–related Z code or any social risk–related Z code specific to food insecurity (Table 2). Across all intervals of the perinatal period, there was no association between Medicaid ACO implementation and the probability of having a Z code for psychosocial circumstances.

**Figure.**
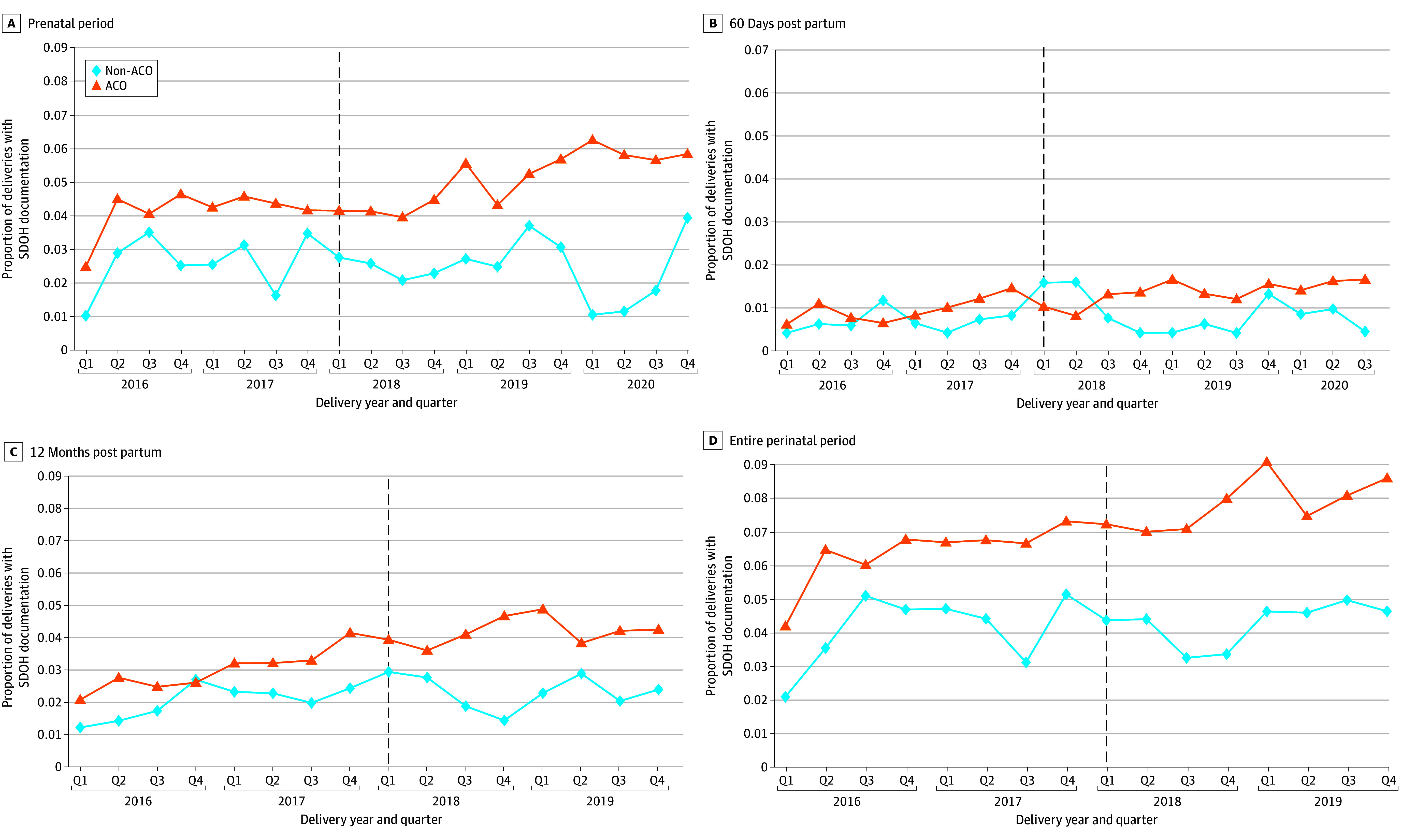
Changes in Claims Documentation of Any Social Risk Factor by Enrollment in a Massachusetts Medicaid Accountable Care Organization (ACO) Graphs present unadjusted quarterly averages. SDOH indicates social determinants of health. The vertical line represents the first quarter of ACO implementation in Massachusetts (2018-Q1).

**Table 2.** Changes in Documentation of Social Risk Factors Associated With the Massachusetts Medicaid ACO, 2016 to 2020

Social risk factor	Medicaid group, %^a^				Difference-in-differences (95% CI), percentage points
	Non-ACO		ACO		
	Preintervention	Postintervention	Preintervention	Postintervention	
**Prenatal period**					
Any	2.60	2.47	4.16	5.09	1.09 (0.38 to 1.80)^b^
Housing or economic circumstances	0.88	0.98	1.12	2.73	1.52 (1.07 to 1.97)^c^
Psychosocial circumstances	1.38	1.16	1.72	1.60	0.12 (−0.39 to 0.63)
Food insecurity	0.05	0.14	0.06	0.72	0.58 (0.42 to 0.73)^c^
All other	0.68	0.60	1.65	1.54	−0.06 (−0.45 to 0.34)
**60 d Post partum**					
Any	0.68	0.86	0.97	1.35	0.16 (−0.24 to 0.55)
Housing or economic circumstances	0.28	0.34	0.31	0.70	0.26 (0.002 to 0.51)^d^
Psychosocial circumstances	0.28	0.28	0.34	0.31	−0.07 (−0.31 to 0.18)
Food insecurity	0.03	0.13	0.04	0.11	−0.05 (−0.17 to 0.08)
All other	0.13	0.34	0.36	0.48	−0.07 (−0.29 to 0.15)
**12 mo Post partum**					
Any	2.04	2.36	3.03	4.21	0.50 (−0.13 to 1.14)
Housing or economic circumstances	0.78	0.94	1.25	2.15	0.50 (0.10 to 0.91)^d^
Psychosocial circumstances	0.96	0.77	1.07	1.01	0.10 (−0.30 to 0.50)
Food insecurity	0.13	0.20	0.16	0.48	0.16 (−0.01 to 0.34)
All other	0.53	0.90	0.93	1.70	0.22 (−0.15 to 0.60)
**Entire perinatal period**					
Any	4.08	4.25	6.39	7.80	1.49 (0.62 to 2.35)^b^
Housing or economic circumstances	1.54	1.82	2.12	4.10	1.88 (1.31 to 2.44)^c^
Psychosocial circumstances	2.07	1.87	2.60	2.43	0.18 (−0.43 to 0.79)
Food insecurity	0.15	0.31	0.21	1.17	0.70 (0.48 to 0.93)^c^
All other	1.69	1.84	3.00	3.41	0.17 (−0.43 to 0.77)

Abbreviation: ACO, accountable care organization.

^a^ Unit of analysis is the delivery quarter. Linear probability models were used and included indicators for enrollment in an ACO, preintervention vs postintervention period, and their interaction (ACO × postintervention), which represents the difference-in-differences. Covariates included age at delivery, delivery type, multiple gestation, documentation of 3 or more clinical comorbidities, and residence in a rural zip code. Models also were adjusted for zip code–level sociodemographic characteristics, included delivery hospital–level fixed effects, and clustered SEs at the individual level. Clinical comorbidities included diagnosis of body mass index (BMI; calculated as the weight in kilograms divided by the height in meters squared) of 25 to 40, BMI of greater than 40, diabetes, hypertension, hyperlipidemia, cardiovascular disease, asthma, major depression, other depression, and anxiety.

^b^ *P* < .01.

^c^ *P* < .001.

^d^ *P* < .05.

### Sensitivity Analyses

Preintervention trends were statistically similar between intervention (ACO) and comparison (non-ACO) groups using data before 2018 (eMethods in Supplement 1). While estimates excluding 2020 data were generally consistent with our main model, albeit attenuated in magnitude, some estimates increased when 2020 data were excluded (eg, food insecurity in the prenatal period, 12 months post partum, and in the perinatal period overall; any social risk factor 60 days post partum; and housing and economic circumstances 12 months post partum) (eTable 3 in Supplement 1).

## Discussion

In this cross-sectional study of all deliveries among Medicaid-enrolled individuals in Massachusetts, claims documentation of social risk–related Z codes was relatively low across the perinatal period. However, Medicaid ACO implementation was associated with a modest increase in social risk–related Z code claims documentation among Medicaid enrollees in the perinatal period, primarily driven by increases in the prenatal period. The largest increases in Z code documentation were related to housing or economic circumstances. This suggests that explicitly requiring social risk factor screening as part of an ACO contract may increase documentation among perinatal populations. However, claims documentation of Z codes during the perinatal period remains low, perhaps suggesting that adoption of social risk factor screening processes may be low or that Z code documentation in claims data may undercount the prevalence of social risk factors when compared with other data sources.^18,23,26^

Our study adds 2 important contributions to the literature: first, to our knowledge, few studies to date have examined social risk–related Z code documentation among pregnant and postpartum people. Our rates were somewhat higher than national rates of social risk–related Z code documentation among pregnant women (1.17%) in an analysis that used 2018 Medicaid claims data.^27^ Second, our study extends previous work evaluating the impact of the Massachusetts Medicaid ACO program on social risk documentation. Prior work has found that the Massachusetts Medicaid ACO was associated with increased primary care visit rates, decreased unplanned admissions for adults with ambulatory care sensitive conditions, and some improved care utilization and quality among postpartum people.^2,28^ Other work found that compared with privately insured pregnant people, Medicaid ACO implementation was associated with decreases in outpatient mental health care in the postpartum period among Medicaid-enrolled people.^29^ We expand this existing work by examining the association of the Medicaid ACO with social risk–related Z code documentation in the prenatal and postpartum periods.

Importantly, we note that our estimates differ from a 2022 study using social risk factor screening questionnaire data from a single large Medicaid ACO in Massachusetts, where approximately 45% of screened individuals reported 1 or more social risk factors.^26^ This difference likely reflects the fact that social risks, while often well captured within other data sources, are underreported within administrative claims data. Other reasons for differences include that the 2022 study used data from all screenings of all attributed Medicaid patients (rather than pregnant and postpartum people) and focused on a single ACO (rather than all 17).^26^ In contrast, our estimates align with those of other studies that have assessed Z codes within claims data (ie, diagnosis codes used for billing),^23,24^ which collectively suggest underuse of Z code documentation. Data from electronic health records may yield higher positive social risk factor rates that more closely reflect true prevalence of risks.^30,31,32^

Our study has 2 key implications for policy and practice. First, the implementation of large-scale, Medicaid-focused care delivery reforms that emphasize addressing patients’ social needs may modestly improve the documentation of social risk factors for Medicaid enrollees, which is a key step to understanding and addressing the social risks of enrollees. While we were unable to empirically disentangle the specific mechanisms driving changes in Z code documentation, increases in documentation among ACO clinicians may stem from multiple concurrent mechanisms related to Massachusetts’ Medicaid ACO design, including the requirement to screen attributed Medicaid patients for social risk factors, inclusion of unstable housing (measured through either Z code or changes in addresses 3 times in a year) in risk adjustment and payment, development of infrastructure to refer patients with positive social risk screening to community-based social services, and the broader integration of health and social needs for attributed Medicaid-enrolled ACO patients. Massachusetts remains one of the few states to integrate social factors to inform their Medicaid payment models.^21^ Other states that are in the process of designing or reforming their Medicaid ACO programs may consider incorporating similar provisions as Massachusetts as a means of improving social risk documentation. However, in doing so, states may wish to better incentivize the inclusion of social risk–related Z codes in claims data—either as a reporting requirement or as a reimbursable code—to maximize the ability to use claims data to understand social risk prevalences. Incentivizing better documentation within claims is especially important given that claims data are often the only statewide, standardized data source for measuring population-level trends in Medicaid.

Second, for state Medicaid programs aiming to understand and intervene in social risk factors and unmet health-related social needs among pregnant and postpartum people, the prenatal period might be a critical time to screen,^7^ as supported by evidence from our present study showing elevated prevalences of social risk factors in the prenatal period. There is also evidence that Medicaid ACOs may increase timely prenatal care initiation^33^ and overall care engagement within the prenatal period.^2^ Taken together with the recommended American College of Obstetricians and Gynecologists visit schedules of every 4 weeks until a gestational age of 28 weeks,^7^ this provides enhanced opportunity for engaging with patients and connecting them to social supports that may help support a healthy pregnancy and infant home environment. Supporting social risk screening during the prenatal period, through Medicaid reimbursement or development of prenatal-specific social risk screening performance metrics, may help support both adoption and documentation of social risk screening during this time.

### Limitations

Our study has several limitations. First, our data lack information on patient race and ethnicity, limiting our ability to examine potential differences in Z code documentation for racially or ethnically minoritized pregnant and postpartum people who face inequitable health outcomes because of structural racism.^1^ Second, our study period includes the COVID-19 pandemic in the postintervention period. Therefore, we conducted sensitivity analyses that exclude 2020. Third, we could not account for variation in screening practices across ACOs or population prevalence of social risk factors. Fourth, while the codes studied in our analysis build on previous studies, they do not capture documentation of social risk factor screening (eg, Healthcare Common Procedure Coding System code G0136, “administration of a standardized, evidence-based social determinants of health risk assessment,” introduced for Medicare billing in 2024), but rather, only capture whether a patient had a positive indication of a risk factor. Finally, we were unable to directly measure characteristics of ACO and non-ACO health systems, but we know based on our patient-level data that on average, non-ACO systems were more likely to be located in rural areas and served fewer total patients; our analytic approach aims to minimize this potential confounding. Further, one major health system did not participate in an ACO, thus improving the exchangeability with the ACO group; however, residual confounding may exist.

## Conclusions

In this cross-sectional study of all live Medicaid-financed deliveries in Massachusetts, we found that claims documentation of social risk–related Z codes was relatively low across the prenatal and postpartum periods. However, Medicaid ACO implementation was associated with increases in Z code documentation, mainly driven by increases in the prenatal period. Thus, implementation of Medicaid ACOs that incorporate efforts to screen for and address patients’ social risks may be one strategy for increasing documentation of social risks among pregnant patients. However, for states implementing Medicaid care delivery models that measure, risk adjust for, or address enrollees’ unmet social needs, this should be paired with additional strategies (eg, reimbursement policies, payment incentives, regulatory reporting requirements) for increasing social risk–related Z code claims documentation rates.

## Notes

Supplement 1. **eMethods.** Statistical Analysis **eTable 1.** Relevant Diagnosis Codes **eTable 2.** Percentage of Deliveries With Documentation of Social Risk Factors by Enrollment in Massachusetts Medicaid Accountable Care Organization (2016-2020 Pooled) **eTable 3.** Sensitivity Analyses **eFigure 1.** Changes in Documentation of Housing and Economic Circumstances **eFigure 2.** Changes in Documentation of Psychosocial Circumstances **eFigure 3.** Changes in Documentation of Food Insecurity

Supplement 2.
Data Sharing Statement
