# Supplementary material for: Medicaid Accountable Care Organization Implementation and Perinatal Claims Documentation of Social Risk Factors
Source: JAMA Netw Open. 2025 Apr 21;8(4):e255999. doi: 10.1001/jamanetworkopen.2025.5999 (PMC12013353; doi:10.1001/jamanetworkopen.2025.5999)
Supplement: Supplement 1. — eMethods. Statistical Analysis eTable 1. Relevant Diagnosis Codes eTable 2. Percentage of Deliveries With Documentation of Social Risk Factors by Enrollment in Massachusetts Medicaid Accountable Care Organization (2016-2020 Pooled) eTable 3. Sensitivity Analyses eFigure 1. Changes in Documentation of Housing and Economic Circumstances eFigure 2. Changes in Documentation of Psychosocial Circumstances eFigure 3. Changes in Documentation of Food Insecurity [file jamanetwopen-e255999-s001.pdf]

Supplementary Online Content

Nguyen KH, Gordon SH, Lim K, Thompson KD, Ncube CN, Cole MB. Medicaid accountable care organization implementation and perinatal claims documentation of social risk factors. *JAMA Netw Open.* 2025;8(4):e255999. doi:10.1001/jamanetworkopen.2025.5999

eMethods. Statistical Analysis
eTable 1. Relevant Diagnosis Codes
eTable 2. Percentage of Deliveries With Documentation of Social Risk Factors by Enrollment in Massachusetts Medicaid Accountable Care Organization (2016-2020 Pooled)
eTable 3. Sensitivity Analyses
eFigure 1. Changes in Documentation of Housing and Economic Circumstances
eFigure 2. Changes in Documentation of Psychosocial Circumstances
eFigure 3. Changes in Documentation of Food Insecurity

This supplementary material has been provided by the authors to give readers additional information about their work.

© 2025 Nguyen KH et al. *JAMA Network Open*.

eMethods. Statistical Analysis

Regression Specification. Our main model specification, for which the unit of analysis was delivery-quarter, included an indicator for ACO (vs. non-ACO), time (pre [2016-2017] vs. post [2018-2020]), and their interaction (ACO x post), which was our coefficient of interest (i.e., difference in differences). We also adjust for Models adjusted for age, delivery type, multiple gestation, documentation of three or more clinical comorbidities, and zip-code-level rurality and sociodemographic characteristics. Models included delivery hospital-level fixed effects, with standard errors clustered at the individual-level to account for repeated deliveries.

Parallel Trends Assumption. We investigated the parallel trends assumption in two ways: (1) we visually inspected unadjusted pre-intervention trends between ACO and non-ACO deliveries; (2) tested the statistical significance of an interaction between ACO and a linear year-quarter specific time trend using only pre-intervention data (i.e., data from 2016 and 2017). For approach (2) we use the same specification as our main model in terms of covariates, fixed effects, and clustering of standard errors. In the table below, we present the estimates for approach (2), where no estimates were statistically significant.

	**Coefficient (95% CI)**	**p-value**
**Prenatal**		
Any Social Risk Factor	0.17 (-0.09, 0.43)	0.196
Housing and Economic Circumstances	0.03 (-0.13, 0.18)	0.741
Psychosocial Circumstances	0.05 (-0.14, 0.24)	0.610
Food Insecurity	0.01 (-0.0, 0.02)	0.059
Other	0.13 (-0.01, 0.28)	0.069
**60 Days Postpartum**		
Any Social Risk Factor	0.06 (-0.09, 0.20)	0.444
Housing and Economic Circumstances	0.02 (-0.05, 0.10)	0.548
Psychosocial Circumstances	-0.02 (-0.12, 0.08)	0.692
Food Insecurity	0.01 (-0.0, 0.02)	0.063
Other	0.07 (-0.01, 0.14)	0.068
**12 Months Postpartum**		
Any Social Risk Factor	-0.02 (-0.25, 0.21)	0.874
Housing and Economic Circumstances	0.01 (-0.13, 0.15)	0.92
Psychosocial Circumstances	0.09 (-0.25, 0.07)	0.267
Food Insecurity	-0.01 (-0.08, 0.06)	0.767
Other	0.05 (-0.07, 0.18)	0.401
**Perinatal Period**		
Any Social Risk Factor	0.11 (-0.21, 0.42)	0.501
Housing and Economic Circumstances	-0.01 (-0.21, 0.20)	0.954
Psychosocial Circumstances	0.04 (-0.18, 0.26)	0.718
Food Insecurity	0.00 (-0.07, 0.07)	0.975
Other	0.06 (-0.16, 0.28)	0.582

© 2025 Nguyen KH et al. *JAMA Network Open*.

eTable 1. Relevant Diagnosis Codes

Z-Code Categories	Z-Codes
Problems related to education and literacy	Z550 Illiteracy and low-level literacy
	Z551 Schooling unavailable and unattainable
	Z552 Failed school examinations
	Z553 Underachievement in school
	Z554 Educational maladjustment and discord with teachers and classmates
	Z558 Other problems related to education and literacy
	Z559 Problems related to education and literacy, unspecified
Problems related to employment and unemployment	Z560 Unemployment, unspecified
	Z561 Change of job
	Z562 Threat of job loss
	Z563 Stressful work schedule
	Z564 Discord with boss and workmates
	Z565 Uncongenial work environment
	Z566 Other physical and mental strain related to work
	Z5681 Sexual harassment on the job
	Z5682 Military deployment status
	Z5689 Other problems related to employment
	Z569 Unspecified problems related to employment
Occupational exposure to risk factors	Z570 Occupational exposure to noise
	Z571 Occupational exposure to radiation
	Z572 Occupational exposure to dust
	Z5731 Occupational exposure to environmental tobacco smoke
	Z5739 Occupational exposure to other air contaminants
	Z574 Occupational exposure to toxic agents in agriculture
	Z575 Occupational exposure to toxic agents in other industries
	Z576 Occupational exposure to extreme temperature
	Z577 Occupational exposure to vibration
	Z578 Occupational exposure to other risk factors
	Z579 Occupational exposure to unspecified risk factor
Problems related to housing and economic circumstances	Z590 Homelessness
	Z591 Inadequate housing
	Z592 Discord with neighbors, lodgers and landlord

© 2025 Nguyen KH et al. *JAMA Network Open*.

Z-Code Categories	Z-Codes
	Z593 Problems related to living in residential institution
	Z594 Lack of adequate food and safe drinking water
	Z595 Extreme poverty
	Z596 Low income
	Z597 Insufficient social insurance and welfare support
	Z598 Other problems related to housing and economic circumstances
	Z599 Problem related to housing and economic circumstances, unspecified
Problems related to social environment	Z600 Problems of adjustment to life-cycle transitions
	Z602 Problems related to living alone
	Z603 Acculturation difficulty
	Z604 Social exclusion and rejection
	Z605 Target of (perceived) adverse discrimination and persecution
	Z608 Other problems related to social environment
	Z609 Problem related to social environment, unspecified
Problems related to upbringing	Z620 Inadequate parental supervision and control
	Z621 Parental overprotection
	Z6221 Child in welfare custody
	Z6222 Institutional upbringing
	Z6229 Other upbringing away from parents
	Z623 Hostility towards and scapegoating of child
	Z626 Inappropriate (excessive) parental pressure
	Z62810 Personal history of physical and sexual abuse in childhood
	Z62811 Personal history of psychological abuse in childhood
	Z62812 Personal history of neglect in childhood
	Z62813 Personal history of forced labor or sexual exploitation in childhood
	Z62819 Personal history of unspecified abuse in childhood
	Z62820 Parent-biological child conflict
	Z62821 Parent-adopted child conflict
	Z62822 Parent-foster child conflict
	Z62890 Parent-child estrangement NEC
	Z62891 Sibling rivalry

© 2025 Nguyen KH et al. *JAMA Network Open*.

Z-Code Categories	Z-Codes
	Z62898 Other specified problems related to upbringing
	Z629 Problem related to upbringing, unspecified
Other problems related to primary support group, including family circumstances	Z630 Problems in relationship with spouse or partner
	Z631 Problems in relationship with in-laws
	Z6331 Absence of family member due to military deployment
	Z6332 Other absence of family member
	Z634 Disappearance and death of family member
	Z635 Disruption of family by separation and divorce
	Z636 Dependent relative needing care at home
	Z6371 Stress on family due to return of family member from military deployment
	Z6372 Alcoholism and drug addiction in family
	Z6379 Other stressful life events affecting family and household
	Z638 Other specified problems related to primary support group
	Z639 Problem related to primary support group, unspecified
Problems related to psychosocial circumstances	Z640 Problems related to unwanted pregnancy
	Z641 Problems related to multiparity
	Z644 Discord with counselors
	Z650 Conviction in civil and criminal proceedings without imprisonment
	Z651 Imprisonment and other incarceration
	Z652 Problems related to release from prison
	Z653 Problems related to other legal circumstances
	Z654 Victim of crime and terrorism
	Z655 Exposure to disaster, war and other hostilities
	Z658 Other specified problems related to psychosocial circumstances
	Z659 Problem related to unspecified psychosocial circumstances

Notes. “Other” category in this analysis include the following categories: Problems related to education and literacy, Problems related to employment and unemployment, Occupational exposure to risk factors, Problems related to social environment, Problems related to upbringing, and Other problems related to primary support group, including family circumstances.

© 2025 Nguyen KH et al. *JAMA Network Open*.

eTable 2. Percentage of Deliveries With Documentation of Social Risk Factors by Enrollment in Massachusetts Medicaid Accountable Care Organization (2016-2020 Pooled)

	Non-ACO	ACO	All
**Prenatal Period, Mean (SD)**			
Housing and Economic Circumstances	0.943 (9.664)	2.094 (14.318)	1.952 (13.835)
Psychosocial Circumstances	1.25 (11.112)	1.648 (12.732)	1.599 (12.544)
Food Insecurity	0.102 (3.200)	0.459 (6.758)	0.415 (6.428)
Other	0.635 (7.946)	1.582 (12.478)	1.465 (12.017)
Any Social Risk Factor	2.521 (15.677)	4.721 (21.210)	4.451 (20.622)
**60 Days Postpartum, Mean (SD)**			
Housing and Economic Circumstances	0.297 (5.444)	0.534 (7.285)	0.504 (7.085)
Psychosocial Circumstances	0.266 (5.155)	0.315 (5.603)	0.309 (5.550)
Food Insecurity	0.082 (2.862)	0.081 (2.837)	0.081 (2.840)
Other	0.287 (5.349)	0.439 (6.608)	0.420 (6.467)
Any Social Risk Factor	0.799 (8.905)	1.192 (10.854)	1.144 (10.634)
**12 Months Postpartum, Mean (SD)**			
Housing and Economic Circumstances	0.769 (8.734)	1.655 (12.759)	1.546 (12.338)
Psychosocial Circumstances	0.051 (2.263)	0.144 (3.790)	0.914 (9.518)
Food Insecurity	0.164 (4.046)	0.308 (5.539)	0.290 (5.378)
Other	0.707 (8.380)	1.299 (11.322)	1.226 (11.004)
Any Social Risk Factor	2.029 (14.100)	3.485 (18.339)	3.305 (17.878)
**Perinatal Period, Mean (SD)**			
Housing and Economic Circumstances	1.518 (12.22)	3.298 (17.858)	3.078 (17.273)
Psychosocial Circumstances	1.783 (13.235)	2.429 (15.395)	2.350 (15.147)
Food Insecurity	0.236 (4.849)	0.738 (8.558)	0.676 (8.194)
Other	1.732 (13.046)	3.230 (17.680)	3.046 (17.184)
Any Social Risk Factor	3.956 (19.493)	7.242 (25.919)	6.838 (25.240)

Notes. Estimates are percentages (on scale 0-100).

© 2025 Nguyen KH et al. *JAMA Network Open*.

eTable 3. Sensitivity Analyses

**Prenatal**	**Main Model**	**Excluding 2020**
	**Coefficient (95% CI)**	**Coefficient (95% CI)**
Any Social Risk Factor	1.09 (0.38, 1.80)**	0.45 (-0.33, 1.23)
Housing and Economic Circumstances	1.52 (1.07, 1.97)***	1.25 (0.74, 1.76)***
Psychosocial Circumstances	0.12 (-0.39, 0.63)	-0.16 (-0.73, 0.41)
Food Insecurity	0.58 (0.42, 0.73)***	0.61 (0.42, 0.80)***
Other	-0.06 (-0.45, 0.34)	-0.23 (-0.65, 0.18)
**60 Days Postpartum**		
Any Social Risk Factor	0.16 (-0.24, 0.55)	0.37 (-0.40, 0.47)
Housing and Economic Circumstances	0.26 (0.00, 0.51)*	0.21 (-0.07, 0.49)
Psychosocial Circumstances	-0.07 (-0.31, 0.18)	-0.10 (-0.37, 0.17)
Food Insecurity	-0.05 (-0.17, 0.08)	-0.07 (-0.22, 0.08)
Other	-0.07 (-0.29, 0.15)	-0.13 -0.38, 0.12)
**12 Months Postpartum**		
Any Social Risk Factor	0.50 (-0.13, 1.14)	0.57 (-0.15, 1.30)
Housing and Economic Circumstances	0.50 (0.10, 0.91)**	0.56 (0.10, 1.03)*
Psychosocial Circumstances	0.10 (-0.30, 0.50)	0.10 (-0.3, 0.55)
Food Insecurity	0.16 (-0.01, 0.34)	0.24 (0.03, 0.44)*
Other	0.22 (-0.15, 0.60)	0.29 (-0.15, 0.73)
**Perinatal Period**		
Any Social Risk Factor	1.49 (0.62, 2.35)**	1.06 (0.10, 2.02)*
Housing and Economic Circumstances	1.88 (1.31, 2.44)***	1.66 (1.02, 2.30)***
Psychosocial Circumstances	0.18 (-0.43, 0.79)	-0.04 (-0.71, 0.64)
Food Insecurity	0.70 (0.48, 0.93)***	0.80 (0.55, 1.06)***
Other	0.17 (-0.43, 0.77)	0.07 (-0.60, 0.73)

Notes. ***p<0.05,**p<0.01, ***p<0.001. Unit of analysis is the delivery-quarter. Linear probability models were used, including indicators for enrollment in an ACO, pre- vs. post-period, and their interaction (ACO x post), which represents the difference-in-differences. Covariates included age at delivery, delivery type, multiple gestation, documentation of three or more clinical comorbidities, and residence in a rural zip code. Models also adjusted for zip-code level sociodemographic characteristics, included delivery hospital-level fixed effects and clustered standard errors at the individual level. Clinical comorbidities included diagnosis of: body mass index [BMI] 25–40 kg/m^2, BMI >40 kg/m2 , diabetes, hypertension, hyperlipidemia, cardiovascular disease, asthma, major depression, other depression, and anxiety.

© 2025 Nguyen KH et al. *JAMA Network Open*.

eFigure 1. Changes in Documentation of Housing and Economic Circumstances

Panel A: Prenatal Period, Medicaid Non-ACO vs. Medicaid ACO

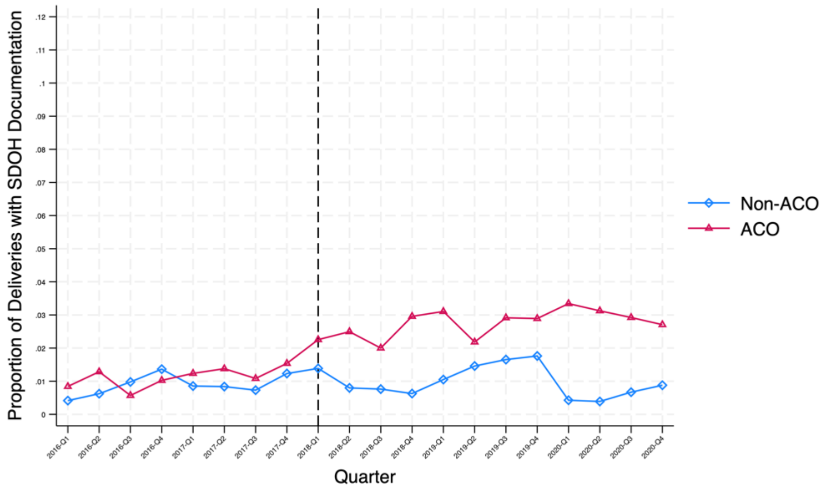

Panel C: 60 Days Postpartum, Medicaid Non-ACO vs. Medicaid ACO

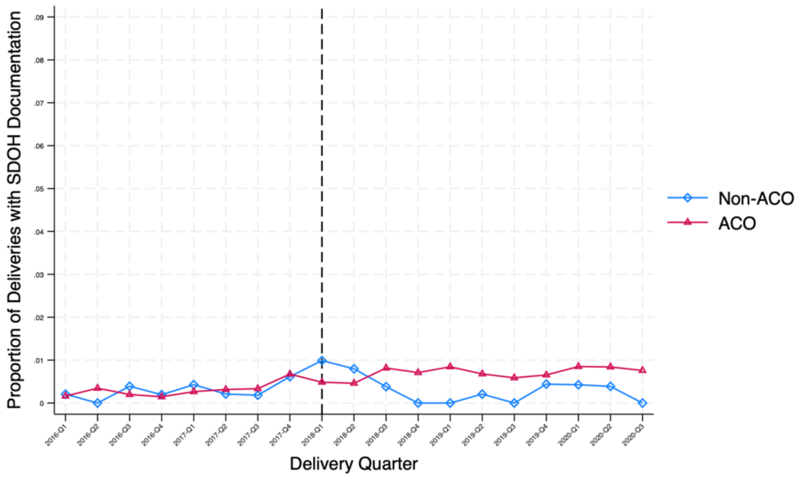

Panel E: 12 Months Postpartum, Medicaid Non-ACO vs. Medicaid ACO

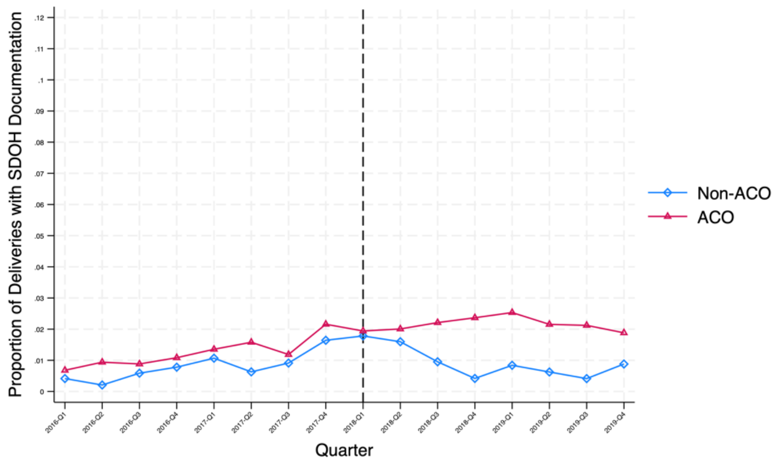

© 2025 Nguyen KH et al. *JAMA Network Open*.

Notes. Graph present unadjusted quarterly averages. SDOH refers to “Social Determinants of Health” and ACO refers to “Accountable Care Organization.” The vertical line represents the first quarter of ACO implementation in Massachusetts (quarter 1 of 2018).

© 2025 Nguyen KH et al. *JAMA Network Open*.

eFigure 2. Changes in Documentation of Psychosocial Circumstances

Panel A: Prenatal Period, Medicaid Non-ACO vs. Medicaid ACO

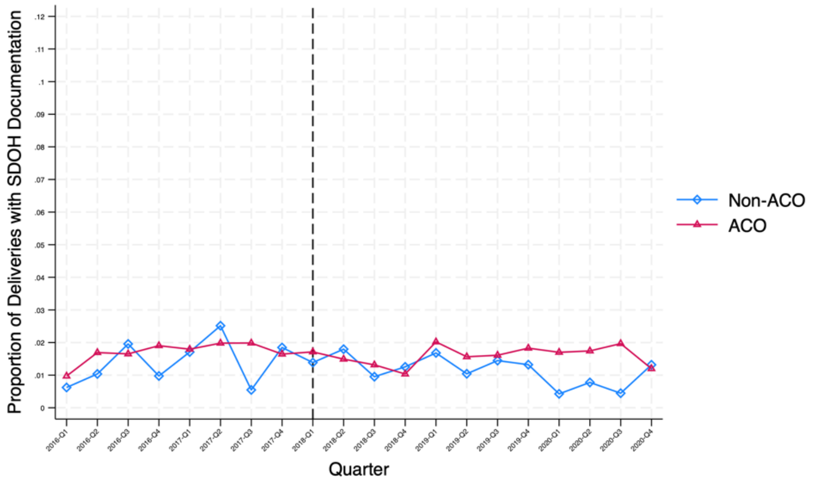

Panel C: 60 Days Postpartum, Medicaid Non-ACO vs. Medicaid ACO

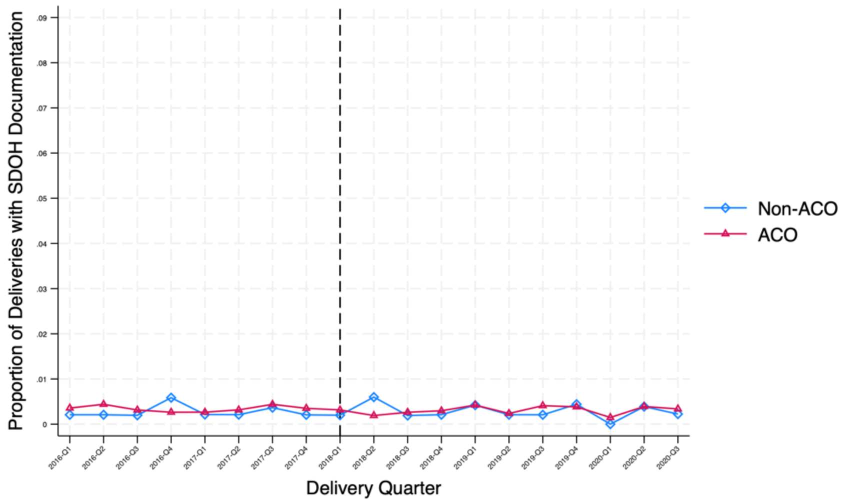

Panel E: 12 Months Postpartum, Medicaid Non-ACO vs. Medicaid ACO

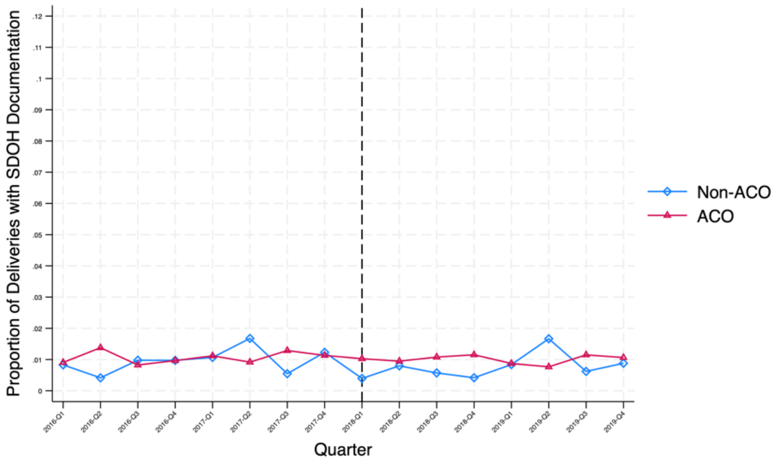

© 2025 Nguyen KH et al. *JAMA Network Open*.

Notes. Graph present unadjusted quarterly averages. SDOH refers to “Social Determinants of Health” and ACO refers to “Accountable Care Organization.” The vertical line represents the first quarter of ACO implementation in Massachusetts (quarter 1 of 2018).

© 2025 Nguyen KH et al. *JAMA Network Open*.

eFigure 3. Changes in Documentation of Food Insecurity

Panel A: Prenatal Period, Medicaid Non-ACO vs. Medicaid ACO

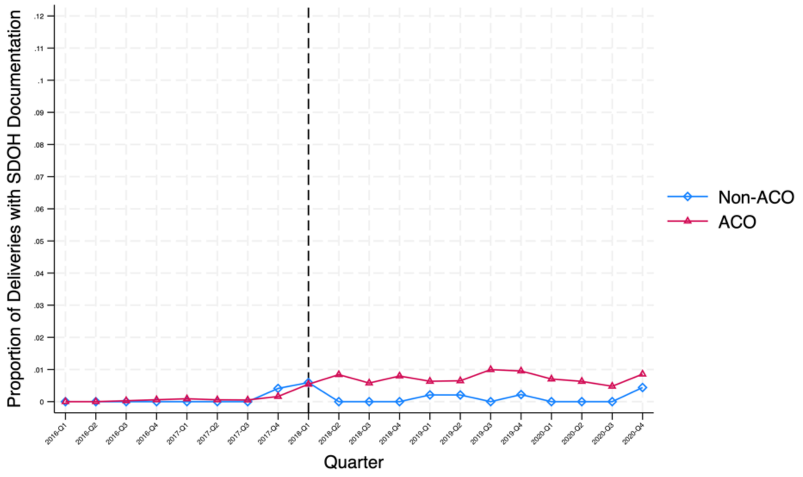

Panel C: 60 Days Postpartum, Medicaid Non-ACO vs. Medicaid ACO

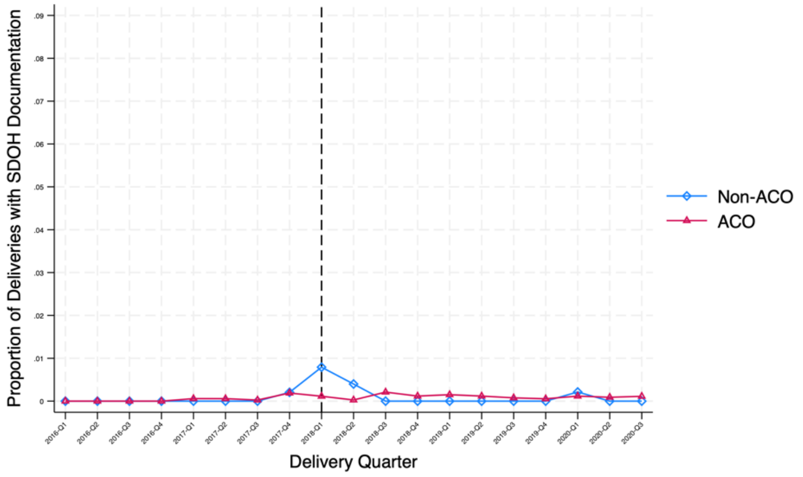

Panel E: 12 Months Postpartum, Medicaid Non-ACO vs. Medicaid ACO

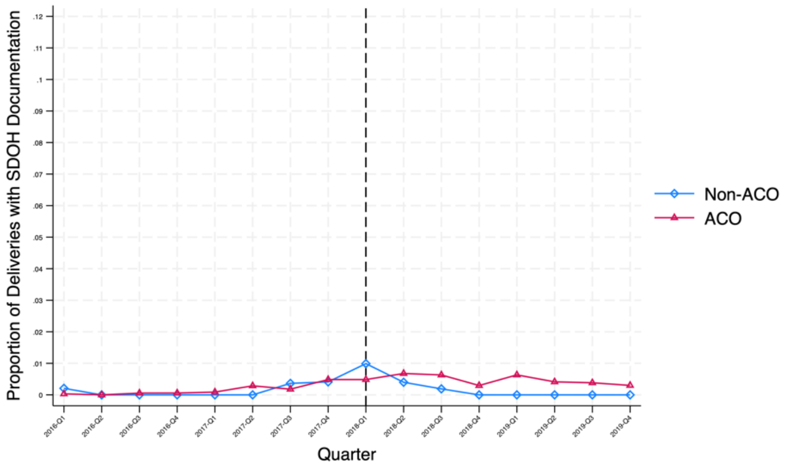

© 2025 Nguyen KH et al. *JAMA Network Open*.

Notes. Graph present unadjusted quarterly averages. SDOH refers to “Social Determinants of Health” and ACO refers to “Accountable Care Organization.” The vertical line represents the first quarter of ACO implementation in Massachusetts (quarter 1 of 2018).

© 2025 Nguyen KH et al. *JAMA Network Open*.
