# Supplementary material for: Medicaid Accountable Care Organization Implementation and Perinatal Claims Documentation of Social Risk Factors
Source: JAMA Netw Open. 2025 Apr 21;8(4):e255999. doi: 10.1001/jamanetworkopen.2025.5999 (PMC12013353; doi:10.1001/jamanetworkopen.2025.5999)
Supplement: Supplement 2. — Data Sharing Statement [file jamanetwopen-e255999-s002.pdf]

Data Sharing Statement

Nguyen. Medicaid Accountable Care Organization Implementation and Perinatal Claims Documentation of Social Risk Factors. *JAMA Netw Open*. Published April 21, 2025. doi:10.1001/jamanetworkopen.2025.5999

Data
Data available: No
